# Administration of benralizumab in a patient with severe asthma admitted to the intensive care unit with COVID-19 pneumonia: case report

**DOI:** 10.1136/ejhpharm-2020-002660

**Published:** 2021-04-21

**Authors:** Johannes Anthon Kroes, Sander Wilhelm Zielhuis, Carina Bethlehem, Anneke Ten Brinke, Eric Nico Van Roon

**Affiliations:** 1 Department of Clinical Pharmacy and Pharmacology, Medical Centre Leeuwarden, Leeuwarden, The Netherlands; 2 Department of Intensive Care, Medical Centre Leeuwarden, Leeuwarden, The Netherlands; 3 Department of Pulmonary Diseases, Medical Centre Leeuwarden, Leeuwarden, The Netherlands; 4 Groningen Research Institute of Pharmacy, Unit of Pharmacotherapy, Epidemiology and Economics, Department of Pharmacy, Rijksuniversiteit Groningen Faculty of Science and Engineering, Groningen, The Netherlands

**Keywords:** COVID-19, pulmonary medicine, safety, case reports, critical care

## Abstract

A patient with severe asthma on benralizumab therapy was admitted to the intensive care unit (ICU) for a coronavirus disease 2019 (COVID-19) infection. At the end of the 8 week benralizumab dosing interval, discussion arose as to whether benralizumab should be administered or if treatment should be discontinued, due to the lack of experience with benralizumab in this situation. Severe broncho-obstruction developed, and the next injection of benralizumab was administered during ICU admission without detrimental symptoms. With this case report, we would like to share our experience with the safe administration of benralizumab during COVID-19 pneumonia, guiding doctors in future decision making.

## Background

To our knowledge, this is the first report of a patient with severe asthma with coronavirus disease 2019 (COVID-19) infection receiving benralizumab during intensive care unit (ICU) admission. The ‘NICE COVID-19 rapid guideline: Severe Asthma’ provides guidelines on biological treatment during the COVID-19 pandemic, but does not mention the use in severe asthma patients on mechanical ventilation.^1^

## Case presentation

A 64-year-old obese woman (body mass index 38.5 kg/m^2^) with severe asthma, for which she had been receiving 8 weekly benralizumab (anti-interleukin-5Rα) injections since December 2019, presented to her general practitioner (GP) with migraine and myalgia. Three days later, she developed a fever and dyspnoea. Azithromycin and a 30 mg/day prednisone course were initiated, but these failed to reduce the signs and symptoms. She was admitted to the hospital with a suspected COVID-19 infection 2 days later. PCR sequencing of the nasopharynx swab was positive for severe acute respiratory syndrome coronavirus 2 (SARS-CoV-2), and a chest x-ray showed bilateral consolidation of the lungs in accordance with severe viral pneumonia. After a day on the isolation ward, her oxygen saturation decreased to 85% and she was admitted to the ICU. The prednisone prescribed by her GP was discontinued at admission.

Initially, at the presentation in the hospital and during the first days of ICU admission, the patient’s asthma was controlled and no broncho-obstructive component was present. However, asthma-related broncho-obstruction developed on the 10th day of ICU admission with hypercapnia (partial pressure of carbon dioxide (PCO_2_) 10.6 kPa), a need for higher oxygen fractions (fractional inspired oxygen (FiO_2_) 50%, arterial oxygen pressure (PaO_2_)/FiO_2_ ratio 126) and an increase in peak airway pressure, but without signs of dynamic hyperinflation. During her stay, the patient received twice daily nebulised budesonide, but this failed to reduce the broncho-obstruction adequately. On day 11 of ICU admission, the 7th week of her 8 week benralizumab dosing interval, 30 mg/day prednisone injections were started and broncho-obstruction decreased over the next days. At the end of the 8 week benralizumab dosing interval, the next benralizumab administration was given at day 17 of ICU admission. The prednisone injections were reduced to 25 mg/day on days 17 and 18, 12.5 mg/day on days 19 and 20, and discontinued on day 21. Airway obstruction decreased 2 days after the benralizumab injection and the patient’s pulmonary status stabilised 4 days after the injection. Eosinophil levels were measured a total of 15 times, but remained undetectable throughout ICU admission. This is in line with early clinical trials, in which the reported benralizumab-induced eosinopenia lasted for at least 8–12 weeks.^2^ Before and during ICU admission, prednisone was administered several times, adding to the observed eosinopenia.

## Treatment

Severe side-effects due to benralizumab have rarely occurred in clinical trials. Therefore, it was decided that the possible benefits of benralizumab treatment outweighed any possible adverse events.

## Outcome and follow-up

The patient tested negative for SARS-CoV-2 by day 31 after which a tracheotomy was performed to facilitate weaning off the mechanical ventilation. She was able to leave the ICU on day 44 to start further physical and mental rehabilitation.

## Discussion

Since there is no evidence that biologics such as benralizumab suppress immunity for viral or bacterial infections, administration during a COVID-19 infection was considered a safe option for this patient with severe broncho-obstruction during mechanical ventilation. Acute hypersensitivity reactions to benralizumab have occurred rarely in clinical trials.^3^ Two recent studies suggested the continuation of biological treatment during the COVID-19 pandemic, but also highlighted the lack of evidence on the subject.^4 5^

Renner *et al* 6 described the consideration concerning biological treatment for eosinophilic asthma during the COVID-19 pandemic and reports two benralizumab-treated severe asthma patients with COVID-19 infection. The COVID-19 infection was very mild in the described cases, as opposed to our case, which described a severe COVID-19 infection.

This is the first case report of benralizumab administration to a severely asthmatic patient on invasive mechanical ventilation. Only two previous cases have reported the administration of biologics in severe asthmatics during ventilation, one reporting the anti-IL-5 drug reslizumab and one reporting the anti-IgE drug omalizumab. In both cases, the administration appeared safe and each patient’s ventilation improved shortly after the administration.^7 8^

**Learning points**

- Phase 2 and 3 trials with benralizumab were conducted in relatively small selected populations. Therefore, reports about the safe administration of benralizumab to specific patients are necessary, increasing the amount of real-world data on benralizumab. Our case adds evidence on the safety of benralizumab administration during ICU admission for a patient with severe COVID-19 pneumonia. Healthcare professionals may consider continuing biological treatment in patients with severe asthma admitted to the ICU with COVID-19 infection.

## Data Availability

All data relevant to the study are included in the article
